# Gambogic Acid and Its Analogs Inhibit Gap Junctional Intercellular Communication

**DOI:** 10.3389/fphar.2018.00814

**Published:** 2018-07-30

**Authors:** Eun J. Choi, Joo H. Yeo, Sei M. Yoon, Jinu Lee

**Affiliations:** ^1^College of Pharmacy, Yonsei Institute of Pharmaceutical Sciences, Yonsei University, Incheon, South Korea; ^2^Department of Integrated OMICS for Biomedical Sciences, Yonsei University, Seoul, South Korea

**Keywords:** gambogic acid, dihydrogambogic acid, tetrahydrogambogic acid, gap junction, connexin, Cx40

## Abstract

Gap junctions (GJs) are intercellular channels composed of connexins. Cellular molecules smaller than 1 kDa can diffuse through GJs by a process termed gap junctional intercellular communication (GJIC), which plays essential roles in various pathological and physiological conditions. Gambogic acid (GA), a major component of a natural yellow dye, has been used as traditional medicine and has been reported to have various therapeutic effects, including an anti-cancer effect. In this study, two different GJ assay methods showed that GA and its analogs inhibited GJIC. The inhibition was rapidly reversible and was not mediated by changes in surface expression or S368 phosphorylation of Cx43, cellular calcium concentration, or redox state. We also developed an assay system to measure the intercellular communication induced by Cx40, Cx30, and Cx43. Dihydrogambogic acid (D-GA) potently inhibited GJIC by Cx40 (IC50 = 5.1 μM), whereas the IC50 value of carbenoxolone, which is known as a broad spectrum GJIC inhibitor, was 105.2 μM. Thus, D-GA can act as a pharmacological tool for the inhibition of Cx40.

## Introduction

Gap junctions (GJs) are intercellular channels that connect the cytosols of contacting cells to allow gap junctional intercellular communication (GJIC). A variety of molecules smaller than 1 kDa, such as nutrients, metabolites, cAMP, Ca^2+^, and inositol trisphosphate (IP_3_), can diffuse through GJs (Alexander and Goldberg, 2003). GJs, which are composed of connexin proteins, are encoded by 21 different genes. A connexon, or hemichannel, is a homo- or hetero-hexamer of connexins. Two connexons of adjacent cells dock with each other to form a GJ (Willecke et al., 2002); subsequently, GJs assemble to form a macromolecular GJ plaque.

The many diseases caused by mutations in connexin-coding genes, including deafness (*Cx26, Cx30, Cx31, Cx32, and Cx43*) and skin disorders (*Cx26, Cx30, and Cx31*), provide evidence that GJs play important roles in normal physiology (Wei et al., 2004). GJs are also involved in the pathophysiology of non-genetic diseases through the “bystander effect.” Toxic hepatitis induced by acetaminophen (Patel et al., 2012), the inflammatory response triggered by Shigella (Kasper et al., 2010), and myocardial necrosis in ischemia-reperfusion (Garcia-Dorado et al., 2004) can be aggravated by GJIC, known as the “kiss of death.” In contrast, the genetic knockout of GJIC exacerbated neuronal damage caused by brain ischemia (Oguro et al., 2001; Ozog et al., 2002), which suggested that GJIC offers a “kiss of life” by spatial buffering of toxic substances or signals. Thus, compounds with an effect on GJIC have therapeutic or toxicological importance.

Gambogic acid was identified as the main component of gamboge, the yellow latex of *Garcinia* species (Ollis et al., 1965). In China and Southeast Asia, GA has long been used for detoxification, homeostasis, and anti-inflammatory and anti-parasitic medicines (Zhang et al., 2014). Recently, GA was reported to be a promising anti-cancer candidate, whose mechanism includes microtubule depolymerization and the phosphorylation of JNK1 and p38 (Chen et al., 2008), inhibition of telomerase (Wu et al., 2004), suppression of vascular endothelial growth factor receptor 2 (Yi et al., 2008), modulation of nuclear factor-kappaB signaling (Pandey et al., 2007), and proteasome inhibition (Li et al., 2013). Also, we identified GA and its analogs, dihydrogambogic acid (D-GA) and tetrahydrogambogic acid (T-GA), as GJIC inhibitors via high-throughput screening. This study aimed to confirm the inhibition, uncover the mode of action, and assess its connexin-selectivity by using the GJIC assay methods developed in this study.

## Materials and Methods

### Chemicals

GA, D-GA, and T-GA were purchased from MicroSource Discovery Systems (Gaylordsville, CT, United States). Epidermal growth factor (EGF), phorbol 12-myristate 13-acetate (PMA), carbenoxolone (CBX), cycloheximide (CHX), and brefeldin A (BFA) were acquired from Sigma-Aldrich (St. Louis, MO, United States).

### Cell Culture

The human glioma cell lines LN215 and *GJA1*-null LN215, and their donor and acceptor cells expressing SLC26A4 and YFP^QL^, respectively (Lee et al., 2015) and the human embryonic kidney cell line HEK293T (ATCC, Manassas, VA, United States) were cultured in Dulbecco’s modified Eagle Medium (DMEM, Welgene, Daegu, South Korea) supplemented with 10% fetal bovine serum, 100 IU/mL penicillin, and 100 μg/mL streptomycin. DMEM/F12 medium supplemented with 10% FBS (Welgene) was used for FRT-Cx43 cells. All animal cells were maintained at 37°C in a humidified atmosphere of 5% CO_2_/95% air.

### Plasmid Construction

To generate the lentiviral transfer plasmids expressing Cx43, Cx40, Cx31, and Cx30, coding sequences were prepared and inserted into the vectors pLVX-EF1α-IRES-Puro (pLVX-EIP, Clontech, Mountain View, CA, United States) or pLVX-CIBla, which was generated from pLVX-EIP through the substitution of the EF1α promoter with a CMV promoter and puromycin acetyltransferase with blasticidin S deaminase. The sequence of multiple cloning sites was 5′-tctaga ggatcc actagt tctaga gcggccgc ggatcc-3′, with the restriction enzyme sites of XbaI, BamHI, SpeI, XbaI, NotI, and BamHI. The plasmid construction information is presented in Supplementary Table 1.

The plasmid expressing SpCas9 and sgRNA targeting *GJA1* was constructed by the insertion of a double-stranded oligonucleotide formed by annealing 5′-CACC G AAT CCT GCT GCT GGG GAC AG-3′ and 5′-AAAC CTG TCC CCA GCA GCA GGA TT C-3′ into the BsmBI site of LentiCRISPRv2 (Addgene #52961). The surrogate reporter plasmid for the enrichment of *GJA1*-null cells was constructed through the introduction of a DNA fragment formed by annealing 5′-AATTC AAT CCT GCT GCT GGG GAC AGC GGT G-3′ and 5′-GATCC ACC GCT GTC CCC AGC AGC AGG ATT G-3′ into the EcoRI/BamHI site of pHRS, kindly provided by Dr. Hyongbum Kim (Ramakrishna et al., 2014).

### Lentivirus Production

HEK293T cells were plated on 6-well plates at a density of 4 × 10^5^ cells/well and grown for 24 h. The transfer plasmid, packaging plasmid (psPAX2, Addgene #12260), and envelope plasmid (pMD2.G, Addgene #12259) were mixed at a ratio of 4:3:1 and 3 μg of the mixture was transfected into HEK293T cells with Lipofectamine 2000 (Invitrogen, Carlsbad, CA, United States) for 15 h. The cells were refreshed with 2 mL of growth medium and cultivated for a further 36 h. The medium containing the lentivirus was harvested, cleared by centrifugation at 3,000 × *g* for 3 min, and stored at -80°C before use.

### Knockout of GJA1 in LN215 Cells Using the CRISPR/Cas9 System

The lentiCRISPRv2 plasmid targeting *GJA1* and the pHRS plasmid containing the same Cas9 target site were mixed at a ratio of 1:1 and electroporated into LN215 cells using a Neon transfection kit (Invitrogen). After incubation for 72 h, *GJA1*-null cells were enriched by selection with 500 μg/mL hygromycin (AG Scientific, San Diego, CA, United States) for 48 h. The surviving cells were plated on a 96-well plate at a final density of 0.5 cells/well (i.e., some wells were empty, while a single cell was placed in the remaining wells) and grown until a sufficient number of cells were obtained. Genomic DNA was extracted from the clonal LN215 cells and used as a PCR template. The DNA fragment encompassing the target site was amplified using the following PCR primer pair: 5′-AGG GAA GGT GTG GCT GTC AGT AC-3′ and 5′-GAT GTA CCA CTG GAT CAG CAA GAA GG-3′ before the T7E1 (NEB, Ipswich, MA, United States) assay, which was conducted to identify clones bearing indels in the intended site of *GJA1*. The PCR products from the identified clones were further analyzed by TA-cloning followed by sequencing. A clone was finally chosen as the *GJA1*-null LN215 cell based on that all 10 read indels were out-of-frame and on its morphological similarity to the original LN215 cells.

### Generation of Cx43-, Cx40-, Cx31-, and Cx30-LN215 Cells

*GJA1*-null LN215 cells were plated on a 24-well plate at 30% confluency, cultivated for 24 h, and transduced with 200 μL of conditioned medium containing lentivirus mixed with an equal volume of fresh growth medium for 15 h. The growth medium was refreshed followed by incubation for a further 72 h before selection with 2 μg/mL puromycin for one week.

### I-YFP GJIC Assay

The I-YFP GJIC assay was conducted as previously reported (Lee et al., 2015, 2016). The scheme of the assay is as follows: donor cells express SLC26A4 as an iodide transporter to allow iodide to the influx and donate iodide to acceptor cells through GJs; the acceptor cells express YFP^QL^ as an iodide sensor, for which the fluorescence is rapidly and sensitively quenched by iodide transferred from donor cells. A 2:1 mixture of the donor (LN215-I) and acceptor (LN215-YFP) cells were plated on 96-well plates at a density of 20,000 cells/well and grown for 24 h. After the medium was removed, the cells were treated with 100 μL of C-solution (10 mM HEPES, pH 7.4, 140 mM NaCl, 10 mM glucose, 5 mM KCl, 1 mM MgCl_2_, 1 mM CaCl_2_) containing vehicle or chemicals, as indicated. A POLARstar microplate reader (BMG Labtech, Ortenberg, Germany) equipped with an automated injector was utilized in the kinetic mode to read the YFP fluorescence of a well every 0.4 s for 10 s. An equal volume of I-solution, with the same composition as C-solution, except for the substitution of NaCl with NaI, was injected into the well 1 s after each recording was started. The % YFP quenching and % GJIC activity were calculated from a previously reported formula (Lee et al., 2016).

### Gap-Fluorescence Recovery After Photobleaching (Gap-FRAP) Assay

The Gap-FRAP assay was performed as previously described (Lee et al., 2016). FRT-Cx43 cells were grown to full confluency in a confocal dish (SPL Life Sciences, Pocheon, South Korea). The cells were loaded with 10 μM calcein-AM (Sigma-Aldrich) in C-solution for 20 min before drug treatment. Cells surrounded by more than four adjacent cells were selected and photobleached using an LSM720 (Zeiss, Jena, Germany). The fluorescent images were collected before and immediately after (0 s) photobleaching, and then at 5 s intervals for 150 s. The percentage of fluorescence recovery was calculated as previously described.

### Analysis of Cx43 With Immunoblot

To assess the cellular level and S368 phosphorylation status of Cx43 on cell surfaces and in whole lysates, LN215-Cx43 cells on 100 mm culture plates were biotinylated using EZ-Link^TM^ Sulfo-NHS-SS-Biotin (ThermoFisher Scientific, Cat. No. 21331) according to the manufacturer’s instructions followed by lysis with PBS containing 1% Triton X-100, 1x protease inhibitor cocktail (Roche), and 1x phosphatase inhibitor cocktail (Pierce). Protein concentration was determined using a BCA assay. To collect surface proteins, 500 μg of protein was bound to 20 μL of NeutrAvidin^TM^ Agarose (Pierce) followed by elution with 2x Laemmli sample buffer. The surface samples and 20 μg of whole lysates were analyzed using immunoblotting as previously described (Lee et al., 2016). To confirm the knockout of GJA1, whole lysates were prepared without surface biotinylation. Anti-total Cx43 (BD Biosciences, 612400) and anti-S369-phospho-Cx43 (Cell Signaling, 3511), anti-Na^+^-K^+^-ATPase (Abcam, ab185065), and anti-actin (Santa Cruz, sc-1615) antibodies were used. The protein band densities were measured using ImageJ software.

### Measurement of DCFH Oxidation

looseness1 LN215 cells were grown to confluency on 96-well plates, washed three times with C-solution, and treated with 100 μL C-solution containing 10 μM 2′–7′-dichlorodihydrofluorescein diacetate (DCFH-DA, Sigma-Aldrich) for 1 h; subsequently, the DCF fluorescence was measured. Chemical treatment was conducted during the DCFH-DA loading period. The DCF fluorescence value, which reflects cellular oxidative stress, was measured using a POLARstar microplate reader using excitation and emission wavelengths of 495 and 520, respectively.

### Statistical Analyses

Statistical analyses were computed using GraphPad Prism 5 software. Student’s *t*-test was performed and a *p*-value of <0.05 was considered to indicate a statistically significant difference. To obtain the IC50, log [compound], and % GJIC inhibition values were fitted into the log [inhibitor] vs. normalized response – variable slope equation.

## Results

### Inhibition of GJIC by GA and Its Analogs

**FIGURE 1:**
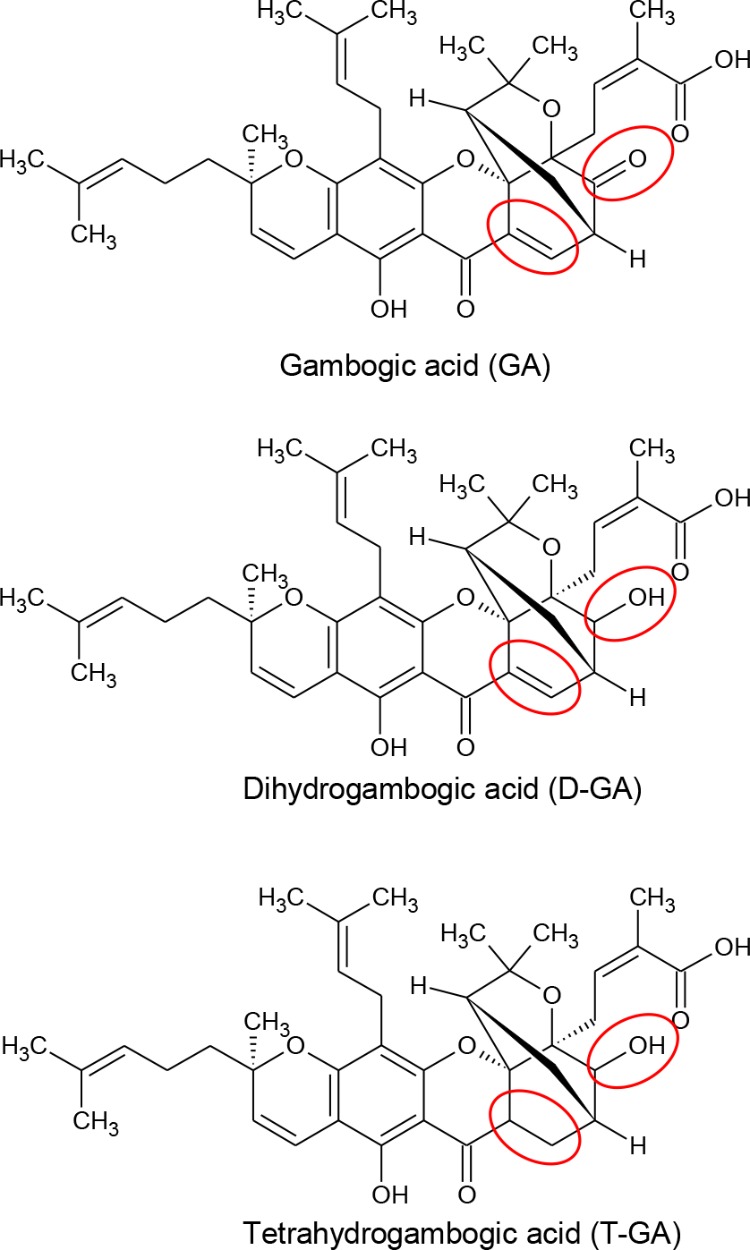
Chemical structures of GA, D-GA, and T-GA. The red circles indicate the structural differences in the three compounds.

**FIGURE 2:**
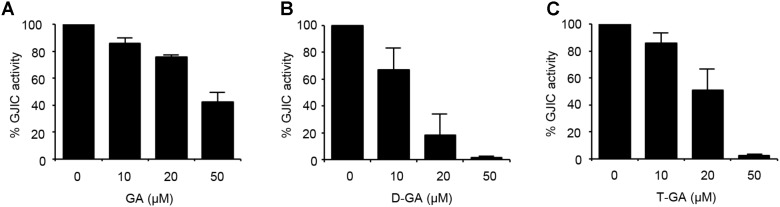
Inhibition of GJIC by GA, D-GA, and T-GA. I-YFP GJIC assay (see Materials and Methods) was conducted using LN215-I and LN215-YFP cells. The vehicle, GA **(A)**, D-GA **(B)**, or T-GA **(C)** was diluted in C-solution and applied to cells at the concentrations indicated above for 10 min before the GJIC assay. The mean % of GJIC activity ± SD of three independent experiments is presented as bar graphs.

To assess the effect of GA, D-GA, and T-GA (**Figure 1**) on GJIC activity, we performed the I-YFP GJIC assay (Lee et al., 2015, 2016) in LN215 glioma cells. As shown in **Figure 2**, GJIC was inhibited by these compounds in a dose-dependent manner. D-GA reduced GJIC to 18.1% ± 15.7% (*n* = 3, mean ± SD) at 20 μM, which was the most potent among them. Thus, most of the subsequent experiments were performed with D-GA.

**FIGURE 3:**
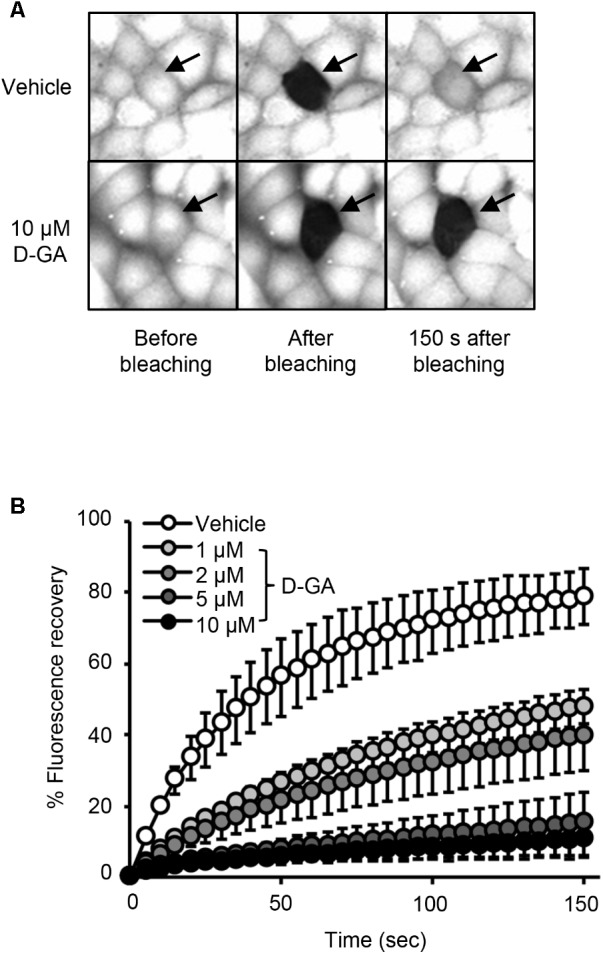
Inhibition of GJIC by D-GA as demonstrated by the Gap-FRAP assay. FRT-Cx43 cells pre-loaded with calcein-AM were treated with vehicle, 1, 2, 5, or 10 μM D-GA before the FRAP assay. Representative images of the gap-FRAP assay are presented in **(A)**. The percentage of fluorescence recovery was plotted as the mean ± SD (*n* = 10) against incubation time after photobleaching **(B)**.

To confirm GJIC inhibition by D-GA, the gap-FRAP assay, a well-known GJIC assay, was conducted in FRT-Cx43 cells. The treatment of the cells with 1, 2, 5, and 10 μM D-GA for 10 min inhibited fluorescence recovery via GJ (**Figure 3**). After photobleaching, the fluorescence at 150 s recovered to 78.7% ± 7.7% (*n* = 10, mean ± SD) and 10.6% ± 5.9% (*n* = 10, mean ± SD) in the vehicle and 10 μM D-GA groups, respectively. The difference in the potency of D-GA between the two assay methods might be due to time lag during the gap-FRAP assay or to the cell difference. When we conducted the gap-FRAP assay, we first found the target cells surrounded by more than four cells, which caused additional incubation. We used FRT-Cx43 cells for the gap-FRAP assay because the cells were more suited for the assay than LN215 cells.

### Reversibility of GJIC Inhibition by D-GA

**FIGURE 4:**
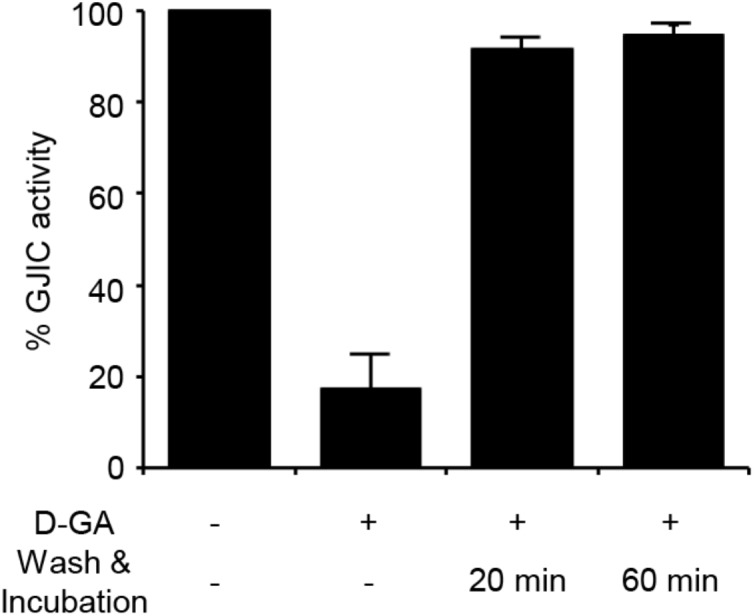
Reversible inhibition of GJIC by D-GA. The 2:1 mixture of LN215-I and LN215-YFP cells were plated on four separate 96-well plates. After incubation for 24 h, the cells were treated as indicated above. D-GA was treated at 20 μM for 10 min. For the wash and incubation groups, the cells were washed with growth media twice and further incubated for 20 or 60 min before the I-YFP GJIC assay was performed. The control group (without D-GA treatment) was used to calculate the percentage of GJIC activity, which was presented as the mean ± SD of three independent experiments in a bar graph.

The I-YFP GJIC assay was performed to examine whether the D-GA-mediated inhibition of GJIC was reversed after the removal of D-GA. The percentage of GJIC activity decreased to 17.6% ± 7.3% after treatment with 20 μM D-GA for 10 min. After washing, incubation for a further 20 min in the absence of D-GA restored GJIC activity to 91.4% ± 2.5% (**Figure 4**).

### D-GA Did Not Alter S368 Phosphorylation of Cx43 but Increased the Surface Expression of Cx43

**FIGURE 5:**
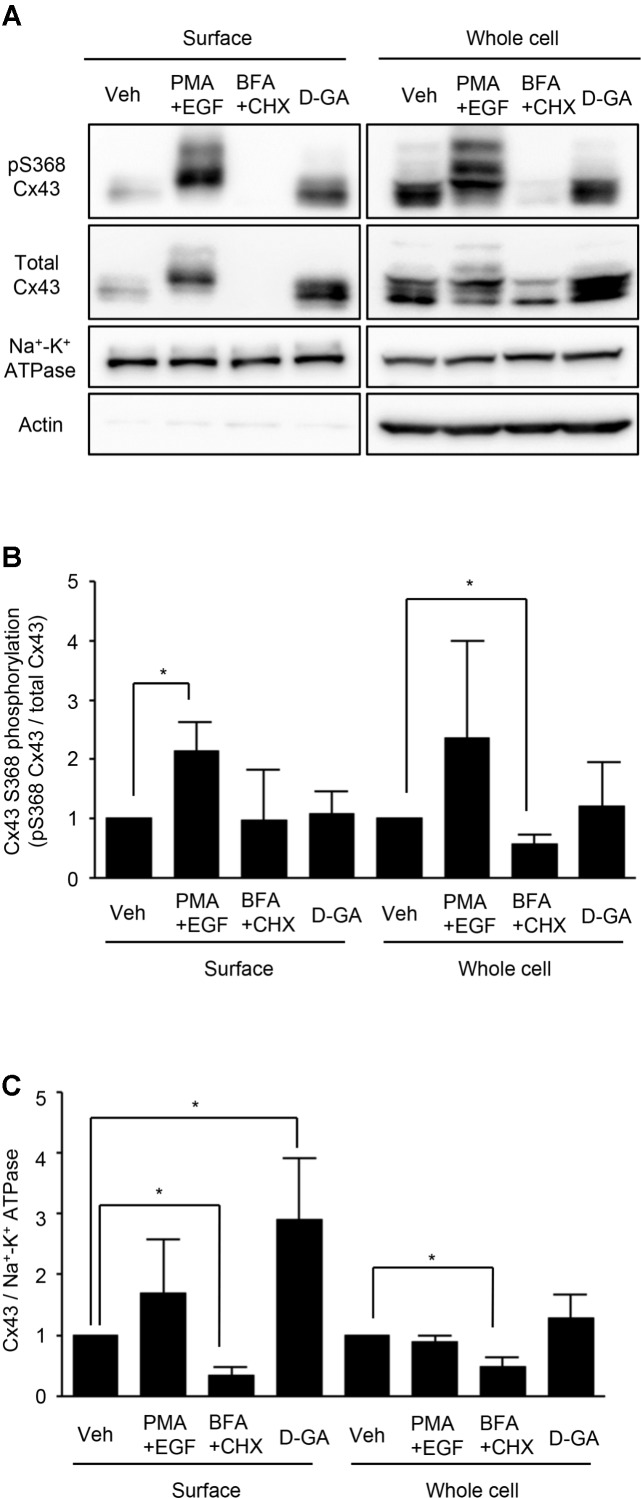
Effect of D-GA treatment on surface expression and S368 phosphorylation of Cx43. LN215-Cx43 cells were grown to 80% confluency in 100 mm plates before treatment with vehicle, 100 ng/mL PMA combined with 50 ng/mL EGF, 1 μg/mL BFA combined with 10 μg/mL CHX or 20 μM D-GA. The treatments were conducted for 10 min except for BFA + CHX, which was treated for 6 h. The whole cell lysates were prepared after surface biotinylation. Biotinylated proteins from 500 μg protein of the whole cell lysates and 20 μg protein of the whole cell lysates were used for immunoblotting of anti-phospho-S368 and total Cx43, anti-Na^+^–K^+^ ATPase, and anti-actin antibodies. Three independent experiments were conducted, and representative blots are presented in **(A)**. The images before cropping are also presented in Supplementary Figures 1A–E. The relative phospho-S368 Cx43 band intensity, divided by the total Cx43 band intensity, is reflective of Cx43 S368-phosphorylation and was calculated and presented as bar graphs **(B)**. The data represent the mean ± SD (*n* = 3). The band intensity of total Cx43 bands was normalized to Na^+^–K^+^ ATPase and presented as a bar graph **(C)**. The data represent the mean ± SD (*n* = 3). ^∗^*P* < 0.05 (Student’s *t*-test); Veh, vehicle.

Connexins located on cell surfaces can form GJs. The effects of D-GA treatment on Cx43 on cell surfaces or in whole cell lysates of LN215-Cx43 cells were analyzed by immunoblotting with an anti-Cx43 and anti-phospho-S368 Cx43 antibodies. The S368 phosphorylation of Cx43 reflects channel closing (Lampe et al., 2000). Cx43 was detected as multiple bands (**Figure 5A**), as shown in a previous report (Brissette et al., 1991). Trace levels of actin were detected from surface protein samples, supporting minimal contamination of cytoplasmic proteins. Whereas co-treatment with EGF and PMA increased S368 phosphorylation of surface Cx43 by twofold, D-GA treatment did not increase S368 phosphorylation, compared with the vehicle-treated group (**Figure 5B**). The cell surface and whole cell levels of Cx43 were significantly reduced by BFA + CHX treatment. The Cx43 level on cell surfaces was increased threefold by D-GA treatment (**Figure 5C**).

### D-GA-Induced GJIC Inhibition Was Not Mediated by Intracellular Calcium Increase or Oxidative Stress

**FIGURE 6:**
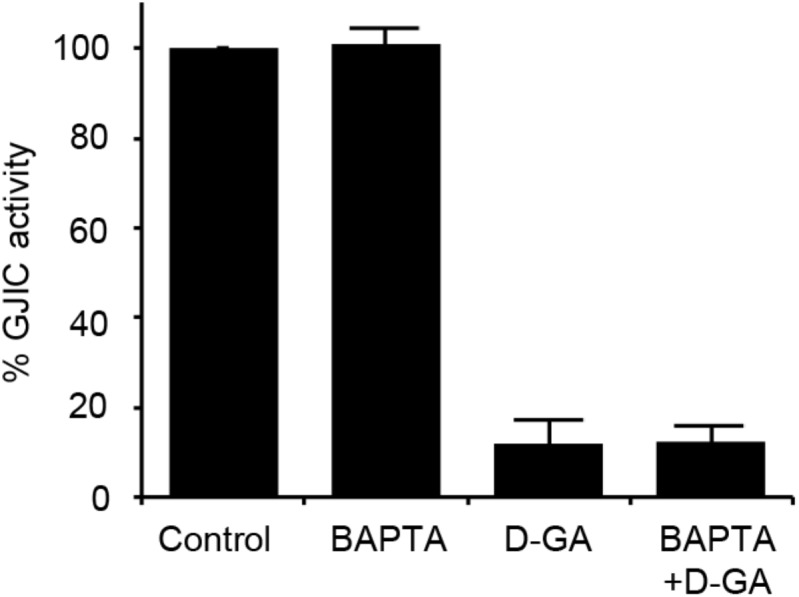
Effect of intracellular Ca^2+^ chelation on GJIC inhibition by D-GA. The LN215-I and LN215-YFP cells were mixed at a 2:1 ratio and plated in 96-well plates. After incubation for 24 h, the cells were pre-treated with vehicle or 5 μM BAPTA-AM in 100 μL of C-solution for 30 min. For the treatment of D-GA at 20 μM, D-GA or its vehicle was added at 20 min of the BAPTA-AM pre-treatment period. Subsequently, the I-YFP GJIC assay was conducted. The data represent the means ± SD (*n* = 3).

As the increase in cellular calcium concentration is a factor that inhibits GJIC (Chen et al., 2008), we examined whether the intracellular chelation of Ca^2+^ with BAPTA-AM attenuated D-GA-induced GJIC inhibition or not. The pre-treatment with BAPTA-AM did not change the GJIC activity in the control or D-GA-treated LN215 cells (**Figure 6**).

**FIGURE 7:**
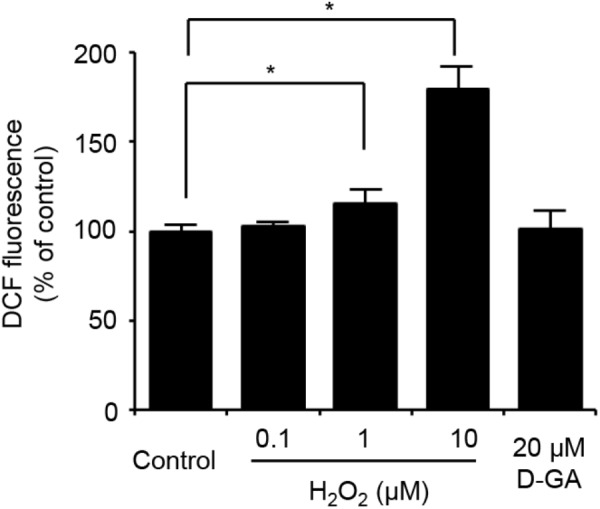
No ROS generation results from D-GA treatment in LN215 cells. LN215 cells were plated in 96-well plates and grown to full confluency. After washing with C-solution, the cells were loaded with 10 μM DCFH-DA diluted in C-solution for 1 h before fluorometry. H_2_O_2_ or D-GA was added after 30 min or 10 min before the measurement, respectively. The data are the mean ± SD (*n* = 3). ^∗^*p* < 0.05 (Student’s *t*-test).

We also assessed the effect of D-GA on the cellular redox state. The cellular production of ROS was measured by using DCF fluorescence. The treatment of LN215 cells with 20 μM D-GA for 10 min did not increase DCF fluorescence, whereas H_2_O_2_ resulted in a dose-dependent increase (**Figure 7**).

### Generation of GJA1-Null LN215 Cell Line

**FIGURE 8:**
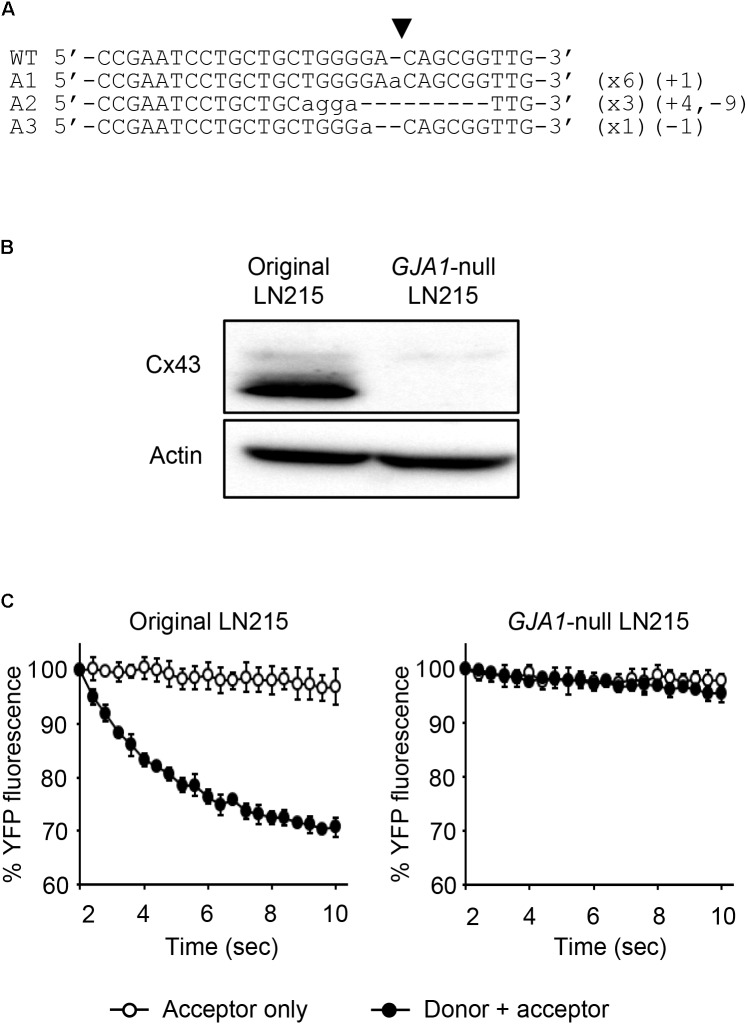
Validation of *GJA1*-null LN215 cells. The genomic DNA sequence alignment of the original and *GJA1*-null LN215 cells in the *GJA1* region targeted by the Cas9 system **(A)**. Ten TA-clones were read, and three types of alleles were identified. Dashes and lower-case letters indicate deleted and inserted residues, respectively. xN, +N, and –N represent the numbers of the TA-clones among the 10 clones inserted, and the deleted bases, respectively. A1, A2, or A3 are three types of alleles. The inverted triangle indicates the cleavage site by Cas9 in *GJA1*. WT, wild-type. The immunoblots showing Cx43 and actin expression of the original and *GJA1*-null LN215 cells **(B)**. The images before cropping are also presented in Supplementary Figures 2A,B. I-YFP GJIC assay using the original (**C**, left) and *GJA1*-null (**C**, right) LN215 cells. The acceptor cells only or the 2:1 mixture of donor and acceptor cells were plated on 96-well plates before the I-YFP assay. The percentage of YFP fluorescence was plotted against assay time. The open and filled circles indicated the acceptor only and donor + acceptor groups, respectively. The data are the mean ± SD (*n* = 3).

To establish the assay systems measuring GJIC made of connexins other than Cx43, we ablated *GJA1* encoding Cx43 in LN215 cells with CRISPR/Cas9 system. Cells bearing indels in the intended site of *GJA1* were enriched by using the surrogate reporter system (Ramakrishna et al., 2014) before cell cloning by limiting dilution. We screened 11 clones in the T7E1 assay and obtained seven clones with the intended indels. The PCR-amplified fragments encompassing the CRISPR/Cas9 target sites from the seven clones were TA-cloned for sequencing. Among them, clone #7 and clone #10 contained out-of-frame mutations in all 10 sequencing results. The original LN215 and clone #10 cells showed similar astrocytic shapes, but clone #7 was polygonal and of no process. The phase contrast images of them are presented in Supplementary Figure 3. Based on the morphological similarity to original LN215 cells, clone #10 was finally selected as the *GJA1*-null LN215 cell line. The alignment of wild-type genomic DNA and read sequences is shown in **Figure 8A**. There appeared to be three copies of *GJA1* in the clone.

The knockout of *GJA1* was confirmed by immunoblotting analysis for the anti-Cx43 antibody. Cx43 was detected in the original LN215 cells, but not in the *GJA1*-null LN215 cells (**Figure 8B**). To examine whether the *GJA1*-null LN215 cells also lost GJIC function, the *GJA1*-null LN215 donor and acceptor cells were generated from the transduction of lentiviruses expressing SLC26A4 and YFP^QL^, respectively. When donor and acceptor cells from original LN215 were used, the I-YFP assay exhibited more rapid YFP quenching in the donor + acceptor group than in the acceptor-only group (**Figure 8C**; left). In contrast, when the *GJA1*-null LN215 donor and acceptor cells were used, there was no significant difference between the acceptor only and donor + acceptor groups (**Figure 8C**; right).

### Measurement of Intercellular Communication by Cx43-, Cx40-, Cx31, or Cx30-GJs

**FIGURE 9:**
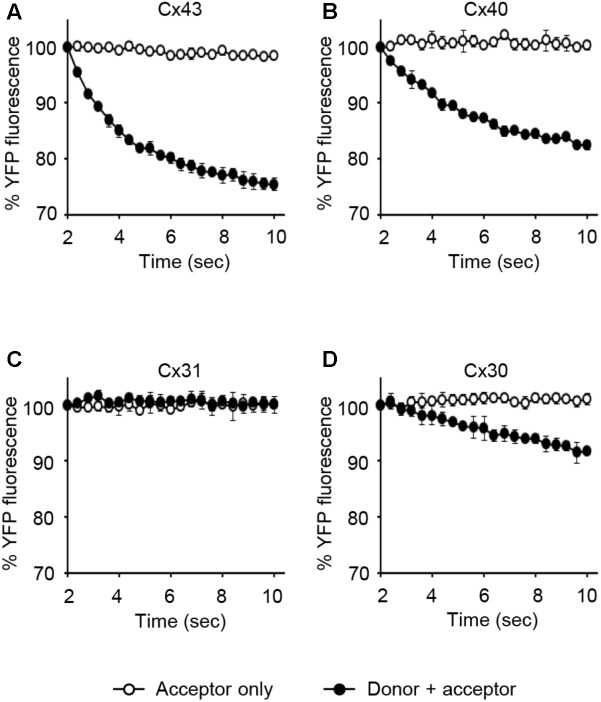
The I-YFP assay measuring GJs composed of Cx43, Cx40, Cx31, or Cx30. The I-YFP GJIC assay was conducted with the acceptor only (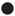) or donor + acceptor cells (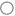) expressing Cx43 **(A)**, Cx40 **(B)**, Cx31 **(C)**, or Cx30 **(D)** (see Materials and Methods for detailed information about the cells) for 10 s. The percentage of YFP fluorescence was plotted against assay time. The data represent the mean ± SD (*n* = 3).

LN215-Cx43, -Cx40, -Cx31, or -Cx30 cells were generated by the transduction of lentivirus expressing corresponding connexins under the CMV promoter. From these cells, donors with SLC26A4 and acceptors with YFP^QL^ were prepared. To examine whether Cx43-, Cx40-, Cx31, or Cx30-GJIC activity could be measured from these cells, acceptors only or a mixture of donor and acceptor cells were plated before the I-YFP assay. GJIC by Cx43, Cx40, and C30 were detected, whereas Cx31-GJIC was not measurable by using this method (**Figure 9**).

### Dose-Response of D-GA Inhibition of Cx43-, Cx40-, and Cx30-GJIC

**FIGURE 10:**
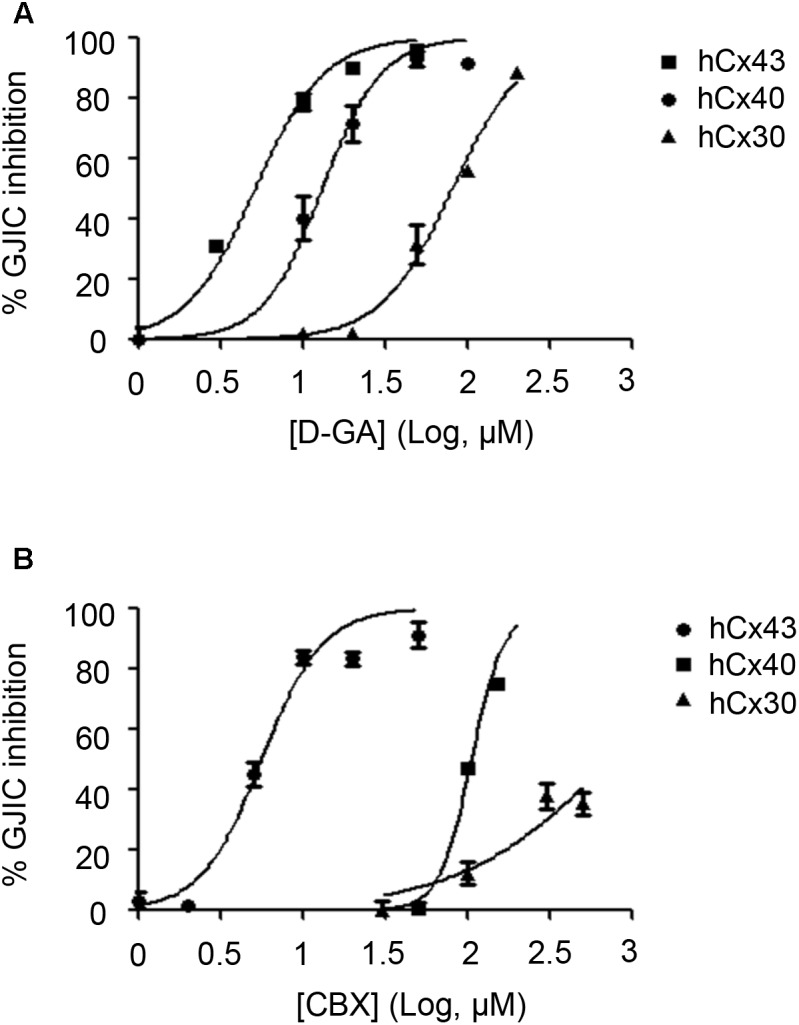
IC50 values of D-GA and CBX inhibition of GJs composed of Cx43, Cx40, or Cx30. The 2:1 mixture of donor and acceptor cells originating from LN215-Cx43, -Cx40, or -Cx30 cells were plated, grown for 24 h, and then treated with various concentrations of D-GA **(A)** or CBX **(B)** for 10 min before the I-YFP assay. The percentage of GJIC inhibition was calculated and plotted against the log [compound] (μM). The data represent the mean ± SD (*n* = 3). The IC50 values were calculated using GraphPad Prism 5.

To assess the connexin-type selectivity of the D-GA-mediated inhibition of GJIC, the dose-response relationships were obtained by using the Cx43-, Cx40-, or Cx30-GJIC assay systems described above. The IC50s of D-GA for the inhibition of GJIC were 13.1 ± 1.1 μM (best fit value ± standard error) for Cx43, 81.1 ± 1.1 μM for Cx30, and, most potently, 5.1 ± 1.1 μM for Cx40 (**Figure 10A**). CBX, a widely-used GJ inhibitor, exhibited the highest potency against Cx43 among the three connexins, with the following IC50 values: 5.6 ± 1.1 μM for Cx43, 105.2 ± 1.0 μM for Cx40, and 748.2 ± 1.3 μM for Cx30 (**Figure 10B**).

## Discussion

In this study, we showed that GA, D-GA, and T-GA inhibited GJIC in LN215 and FRT cells using the I-YFP GJIC assay and the gap-FRAP assay. When the I-YFP assay is used, if a compound inhibits SLC26A4, it can be identified as a GJ inhibitor. GA and its analogs did not inhibit SLC26A4 whereas a known SLC26A4 inhibitor, PDS_inh_-C01 potently inhibited (Supplementary Figure 4). As D-GA was the most potent, subsequent experiments were conducted using D-GA. The GJIC inhibition by D-GA was nearly completely reversed after washing and further incubation in the absence of the compound for 20 min, which suggests that the inhibition did not result from cellular toxicity. The known indirect mechanisms that mediate GJIC inhibition include a decrease in the cellular, especially the cell surface level of Cx43 (Leithe et al., 2010), the phosphorylation of Cx43 (Lampe, 1994; Kwak et al., 1995), the elevation of [Ca^2+^]_in_ (Peracchia, 2004), and ROS generation (Upham et al., 1997). None of these mechanisms were associated with D-GA-induced GJIC inhibition. The surface level of Cx43 was somewhat increased by D-GA treatment for 10 min, but this effect did not explain the inhibition of GJIC and was just an epiphenomenon. The Cx43 increase can be attributed to the proteasomal inhibition by GA (Li et al., 2013). These results suggest a direct inhibition of GJIC by D-GA, such as blocking or closing the GJ channel pore via its binding to connexins. However, electrophysiological methods with a greater time resolution or a direct binding study are needed to obtain more reliable evidence (Verselis and Srinivas, 2013).

To establish a GJIC assay composed of connexins other than Cx43, a putative major connexin expressed in naïve LN215, we first generated *GJA1*-null LN215 cells using a CRISPR/Cas9 technique. Whether a type of cell possesses functional GJs or not is not only dependent on cellular connexin expression, but also on other cellular features (Musil et al., 1990), and we already knew that LN215 cells are suitable for the I-YFP GJIC assay. The genetic knockout of *GJA1* led to the complete functional loss of GJIC, supporting the hypothesis that Cx43 is a major connexin in LN215 cells. The donor and acceptor cells expressing Cx43, Cx40, Cx31, or Cx30 in the I-YFP GJIC assay were generated using lentiviral transduction. Regarding Cx43, Cx40, and Cx30, the assay systems functioned well. Cx31-GJIC was not measured in this system. We measured the IC50 values of D-GA and CBX for the inhibition of Cx43-, Cx40-, or Cx30-GJIC. Interestingly, the IC50s of D-GA and CBX on Cx40 were 5.1 and 105.2 μM, respectively. This remarkable difference was unexpected because CBX was believed to inhibit connexin-channels non-specifically (Verselis and Srinivas, 2013). Furthermore, CBX showed the least potency to Cx30. To the best of our knowledge, D-GA is the only known compound to exert the selective inhibition of human Cx40-GJIC, although relatively.

Cx40 is expressed predominantly in the vascular endothelium and electro-conduction system of the heart (Bastide et al., 1993) and plays essential roles in the regulation of blood pressure (Krattinger et al., 2007; Wagner et al., 2007). Cx40 transgenic mice have been reported as hypertension animal models (Krattinger et al., 2007; Wagner et al., 2007; Morton et al., 2015). Cx40 also regulates platelet function (Vaiyapuri et al., 2013). Based on our results, GA and its analogs can be utilized as a pharmacological tool for the study of the functions of Cx40. The effect of the compounds on various other connexins remains to be evaluated.

## Author Contributions

EC and JL designed the study and prepared the manuscript. EC conducted most of the experiments. JY and SY prepared some cell lines and materials.

## Conflict of Interest Statement

The authors declare that the research was conducted in the absence of any commercial or financial relationships that could be construed as a potential conflict of interest. The reviewer HN and handling Editor declared their shared affiliation.

## Abbreviations

CRISPR: clustered regularly interspaced short palindromic repeats
D-GA: dihydrogambogic acid
GA: gambogic acid
GJ: gap junction
GJIC: gap junctional intercellular communication
T-GA: tetrahydrogambogic acid
