# Supplementary material for: Gambogic Acid and Its Analogs Inhibit Gap Junctional Intercellular Communication
Source: Front Pharmacol. 2018 Jul 30;9:814. doi: 10.3389/fphar.2018.00814 (PMC6077758; doi:10.3389/fphar.2018.00814)
Supplement: Supplementary file 1 [file Table_1.docx]

## Supplementary Table 1. Methods for the preparation of lentiviral plasmids expressing Cx43, Cx40, Cx31, or Cx30.

| Plasmid | Enzyme site | Preparation of insert |
| --- | --- | --- |
| pLVX-CIBla-Cx43 | XbaI | Amplified by nested PCR from human brain cDNA using the following primers: AGG AAA GTA CCA AAC AGC AG and ATA AGG CTG TTG AGT ACC ACC for outer PCR; GC TCT AGA ACC ATG GGT GAC TGG AGC GC and GC TCT AGA CTA GAT CTC CAG GTC ATC AGG C for inner PCR and digested with XbaI |
| pLVX-EIBla-Cx40 | EcoRI/XbaI | Excised from pOTB7-Cx40 (provided from Korea Human Gene Bank, Medical Genomics Research center, KRIBB, Korea) with EcoRI and XbaI |
| pLVX-CIBla-Cx31 | XbaI | Amplified by nested PCR from human genomic DNA using the following primers: CAT GGA CTG GAA GAC ACT C and TTG CAT AAC TTA GTG AAC TCA G for outer PCR; GC TCT AGA ACC ATG GAC TGG AAG ACA CTC and GC TCT AGA TCA GAT GGG GGT CAG GTT G for inner PCR and digested with XbaI |
| pLVX-CIBla-Cx30 | XbaI | Amplified with nested PCR from human brain cDNA using the following primers: TCT TGC CCT CAT TCT TTG and TCT CCT TAT GAC GCA GC TAC for outer PCR; GC TCT AGA ACC ATG GAT TGG GGG ACG C and GC TCT AGA AAA TGT TTA GCT TGG GAA ACC for inner PCR and digested with XbaI |
