# Supplementary figures and images for: Gambogic Acid and Its Analogs Inhibit Gap Junctional Intercellular Communication
Source: Front Pharmacol. 2018 Jul 30;9:814. doi: 10.3389/fphar.2018.00814 (PMC6077758; doi:10.3389/fphar.2018.00814)

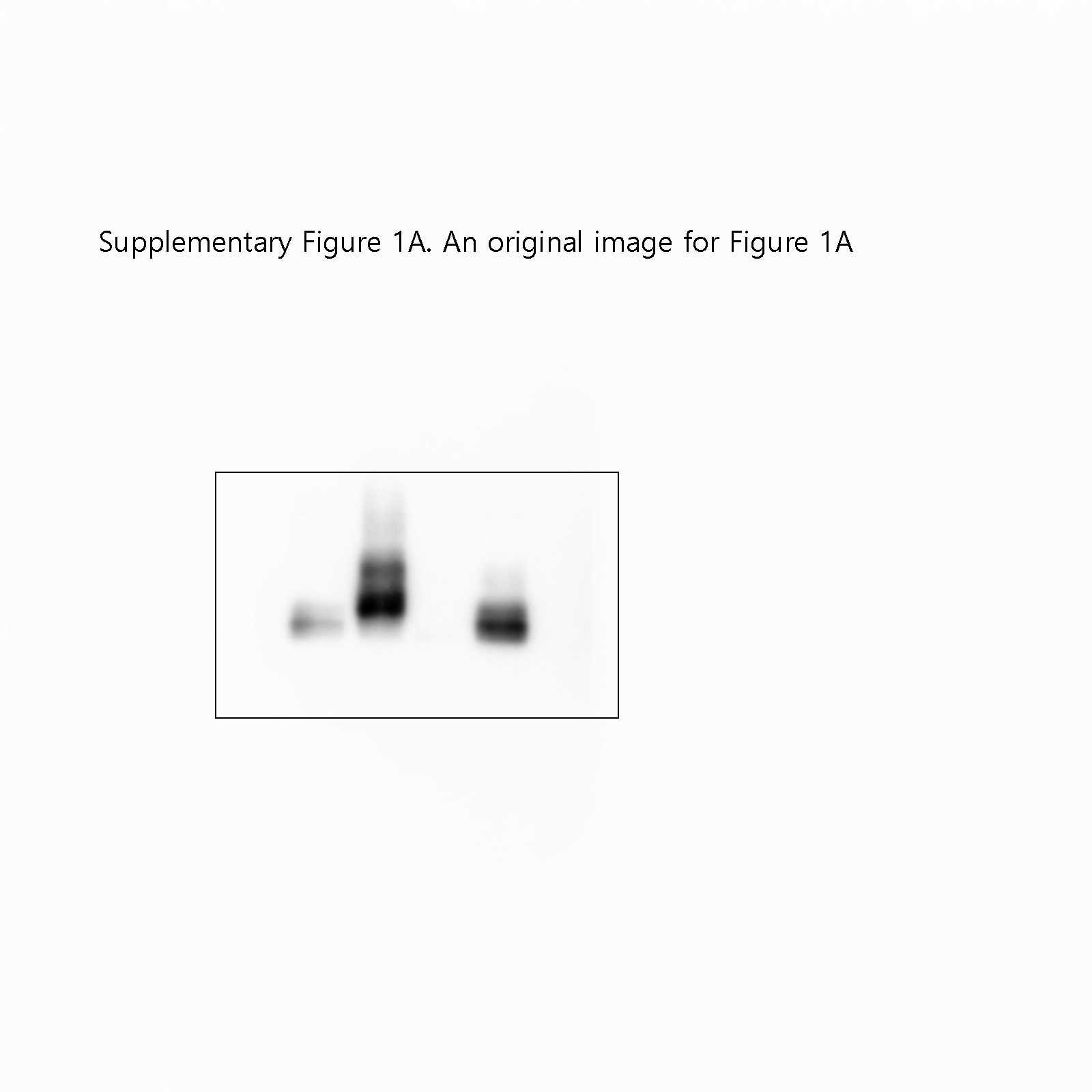

Supplement: Supplementary file 2 [file Image_1.tif]

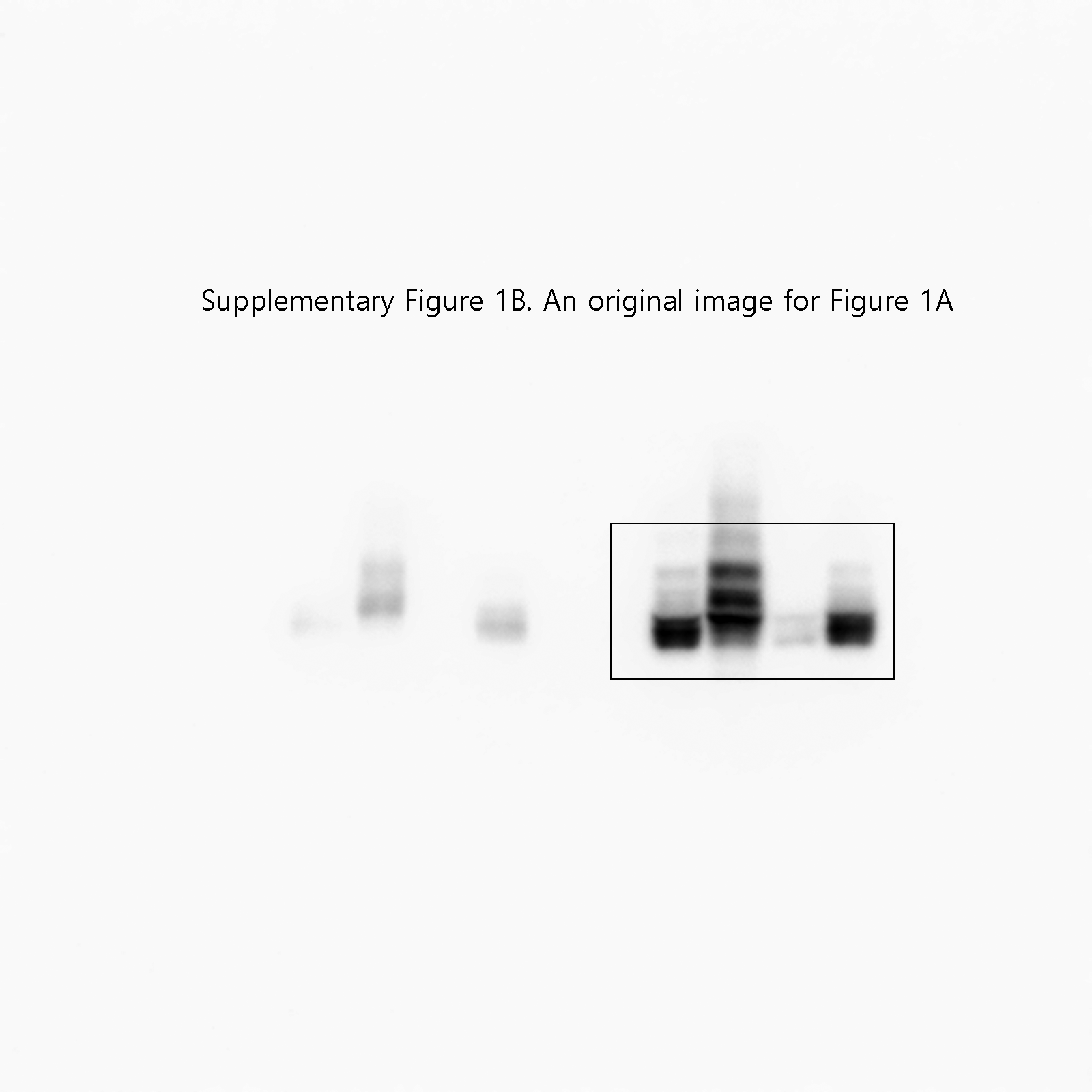

Supplement: Supplementary file 3 [file Image_2.tif]

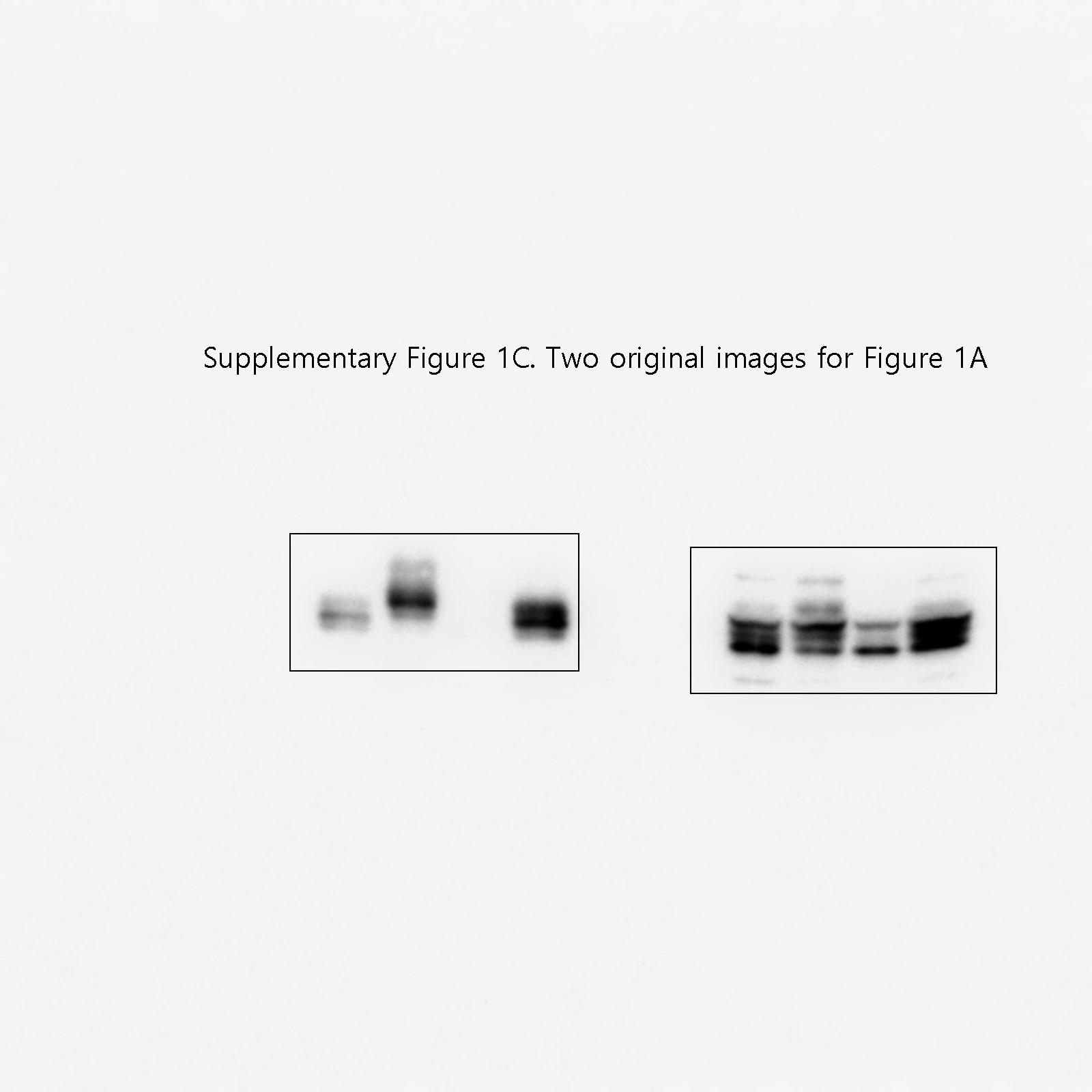

Supplement: Supplementary file 4 [file Image_3.tif]

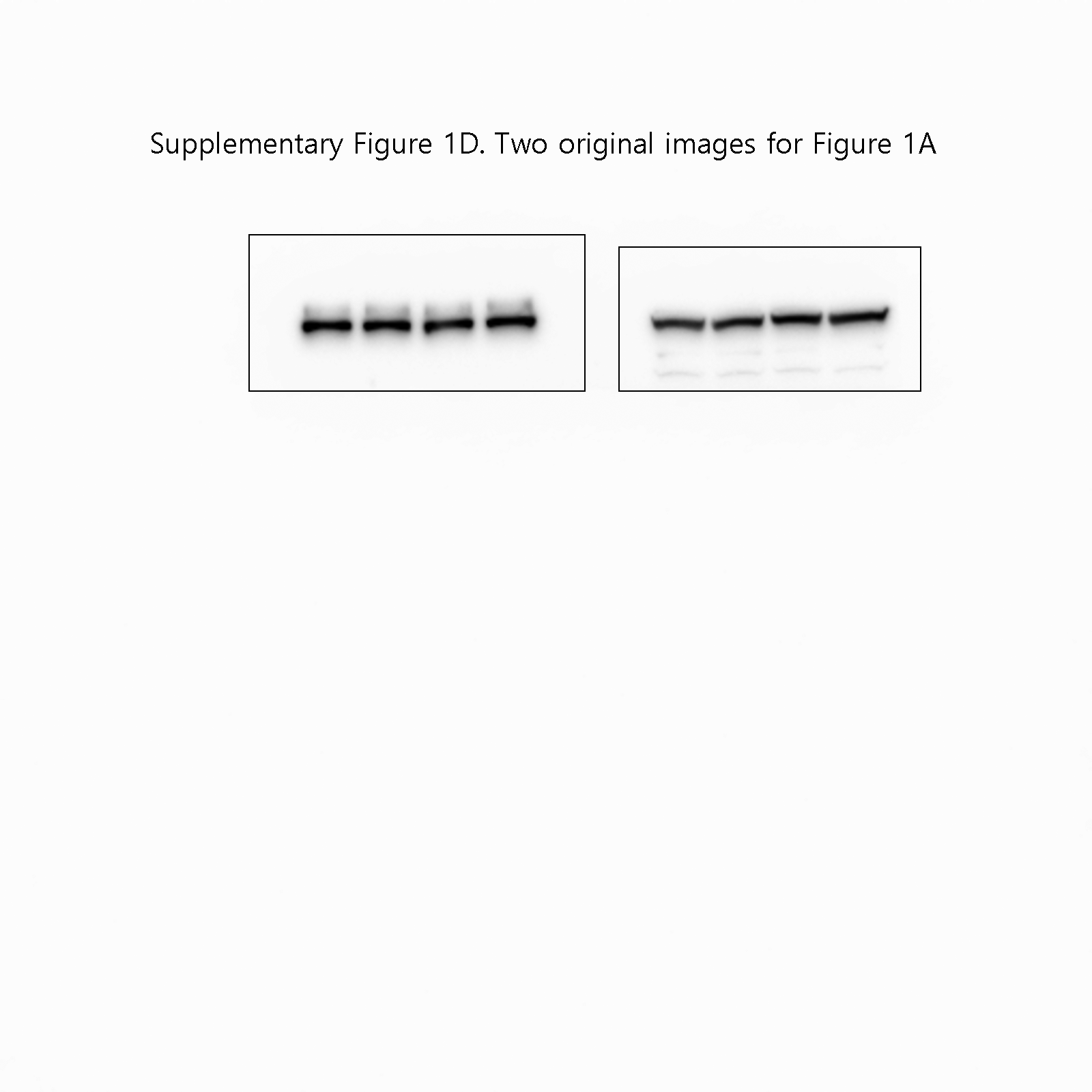

Supplement: Supplementary file 5 [file Image_4.tif]

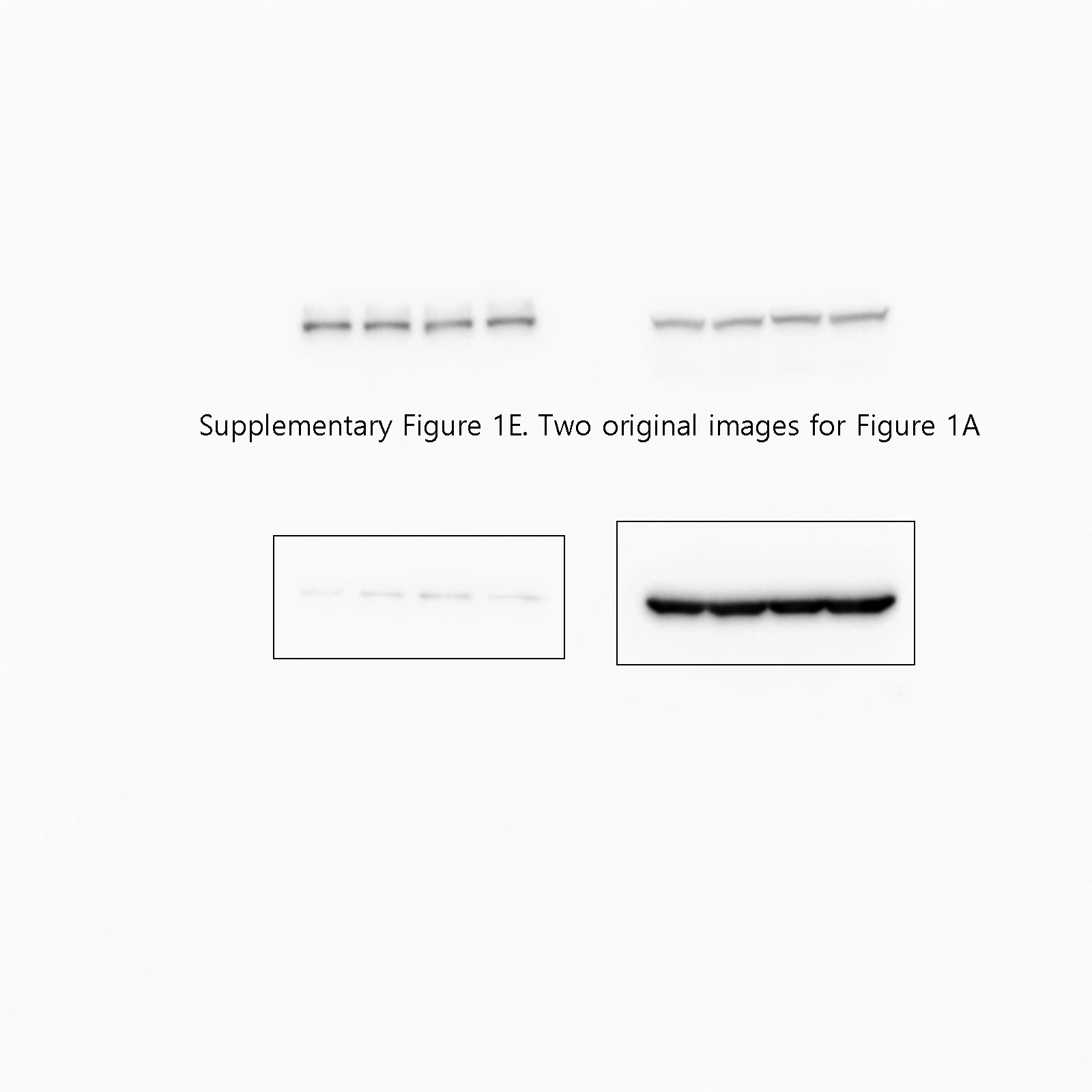

Supplement: Supplementary file 6 [file Image_5.tif]

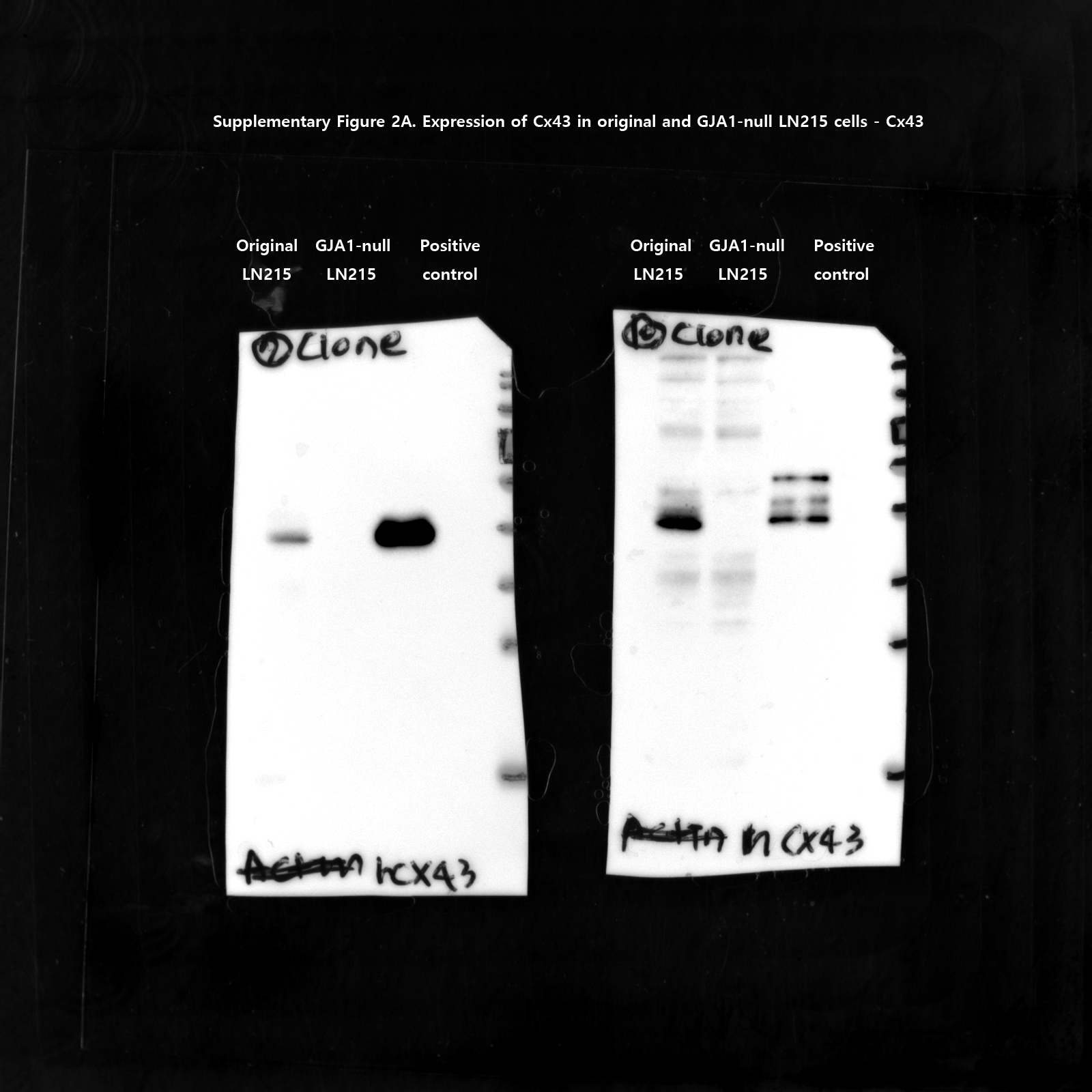

Supplement: Supplementary file 7 [file Image_6.TIF]

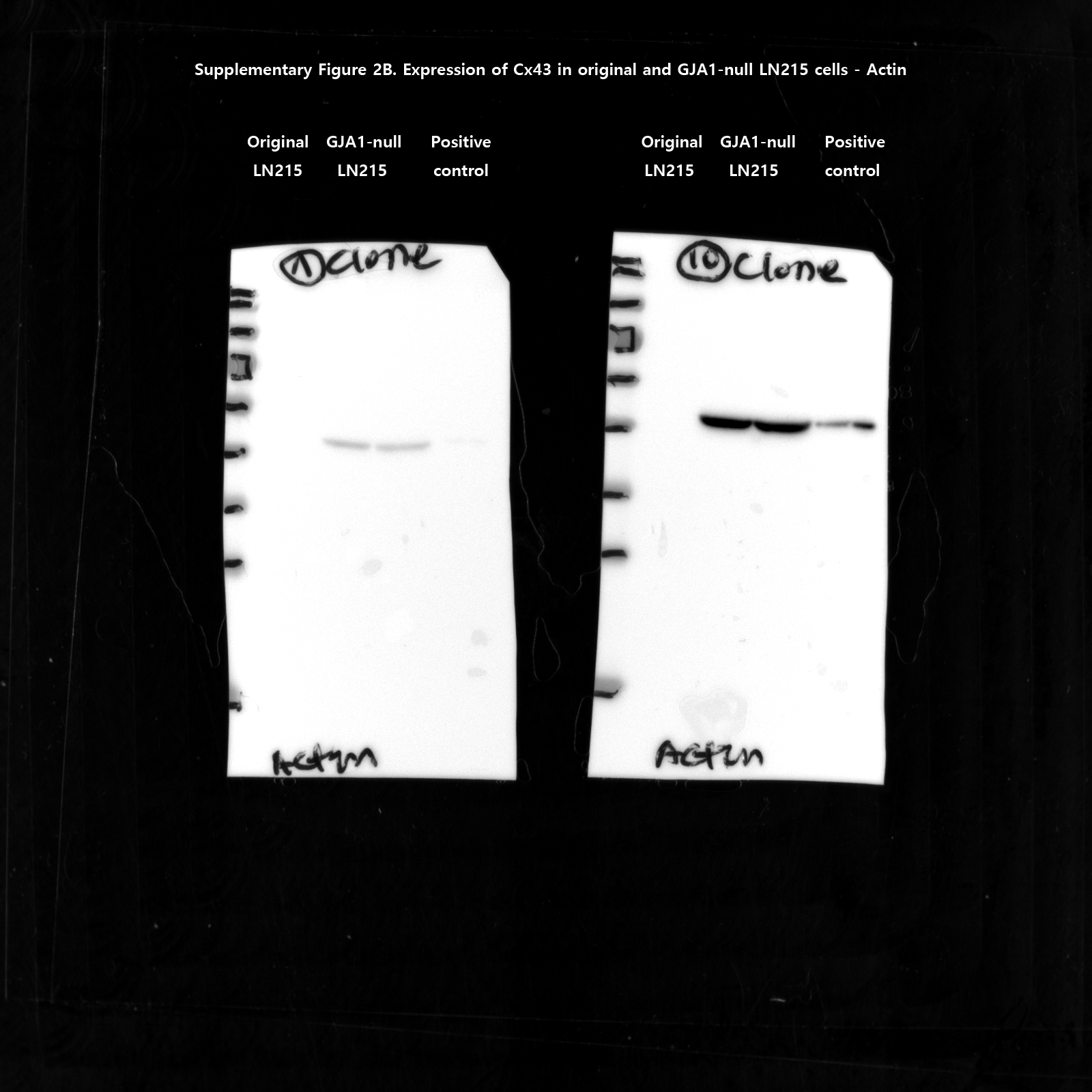

Supplement: Supplementary file 8 [file Image_7.TIF]

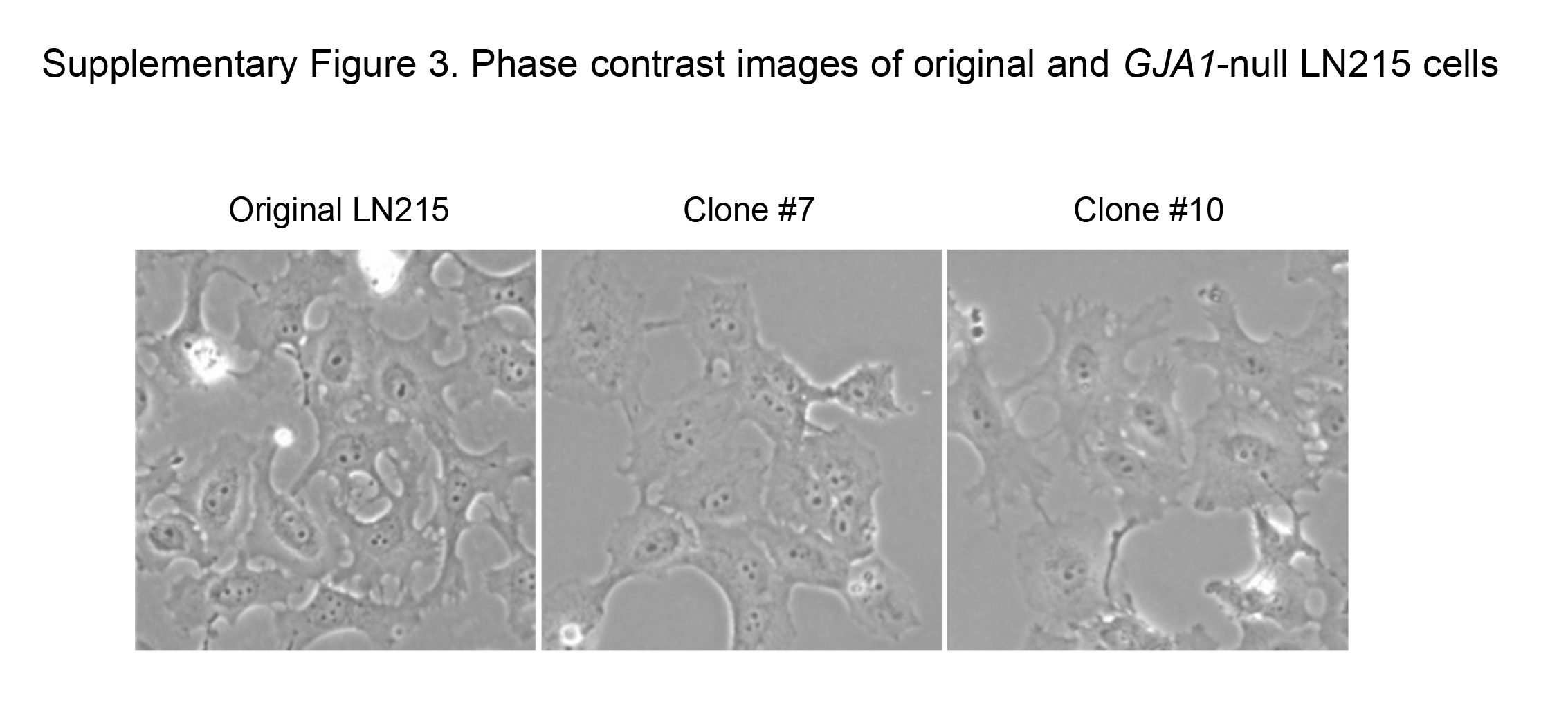

Supplement: Supplementary file 9 [file Image_8.TIF]

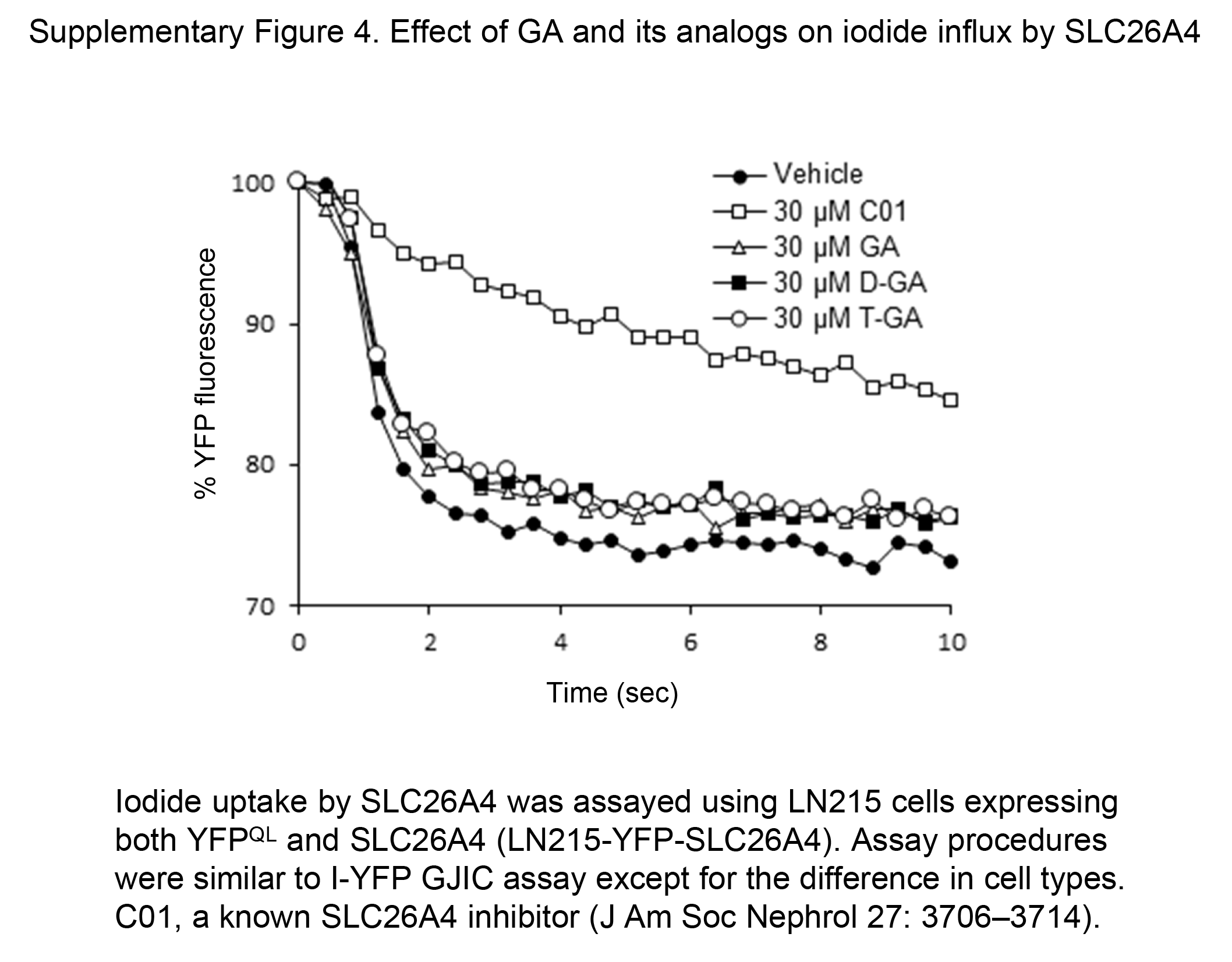

Supplement: Supplementary file 10 [file Image_9.TIF]
